# Targeting TIM-3 for hematological malignancy: latest updates from the 2022 ASH annual meeting

**DOI:** 10.1186/s40164-023-00421-2

**Published:** 2023-07-19

**Authors:** Jiaxiong Tan, Huo Tan, Yangqiu Li

**Affiliations:** 1 Department of Hematology, The Fifth Affiliated Hospital of Guangzhou Medical University, 510700 Guangzhou, China; 2 Institute of Hematology, School of Medicine, Key Laboratory for Regenerative Medicine of Ministry of Education, Jinan University, 510632 Guangzhou, China

**Keywords:** TIM-3, Gal-9, Immunotherapy, Hematological malignancies

## Abstract

T cell immunoglobulin domain and mucin domain-3 (TIM-3) is an important immune checkpoint (IC) protein in cancer immunosuppression that is considered a novel target for immunotherapy. Moreover, TIM-3, an immuno-myeloid regulator, is highly expressed on the cells of several solid tumors and myeloid leukemia stem cells (LSCs). TIM-3 blockade was shown to have dual effects for directly inhibiting leukemia cells and restoring T cell activation. We summarize several of the latest reports on the role of TIM-3 in immunotherapy for hematological malignancies from the 2022 ASH Annual Meeting (ASH2022).

To the editor,

T cell immunoglobulin domain and mucin domain-3 (TIM-3), is significantly expressed on the T cells of cancer and leukemia patients and has been discovered to be associated with tumor progression [1]. Moreover, malignant cells can also express TIM-3, and it is preferentially expressed on myeloid leukemia stem cells (LSCs) [2]. Thus, TIM-3 is not only considered a T cell exhaustion molecule but also a potential target for myeloid cells as an immuno-myeloid regulator. Anti-TIM-3 antibodies have been shown to have efficacy and be safe for the treatment of advanced solid tumors. Recently, TIM-3 inhibitors have been used to treat acute myeloid leukemia (AML) and myelodysplastic syndromes (MDS) in clinical trials. Here, we summarize several of the latest reports on the role of TIM-3 in immunotherapy for HMs from the 2022 ASH Annual Meeting (*A*SH2022).

## Influence of CAR-T cell activation and BiTE function

Chimeric antigen receptor (CAR) –T cell exhaustion or senescence in vivo remains a major issue. Increased expression of immune checkpoint proteins, such as that of TIM-3 during CART19-28ζ cell exhaustion, results in a decrease in cytotoxicity [3]. Moreover, CART19-BBζ cells are more prone to developing a senescent phenotype compared to CART19-28ζ cells [4].

Bispecific antibodies, such as CD19-CD3 T cell engagers (BiTE), have been used for B cell malignancy immunotherapy [5]. In ASH2022, the multicohort, open-label, phase 1/2 MajesTEC-1 study, which investigated the safety/efficacy of teclistamab (B-cell maturation antigen (BCMA)-CD3 bispecific IgG4 antibody) in patients with relapsed/refractory multiple myeloma (RRMM), demonstrated encouraging efficacy. Lower T cell numbers and a higher frequency of T cells expressing IC markers, including TIM-3, are underlying reasons for non-responders with unfavorable immune characteristics at baseline [6]. Overall, the above studies are consistent with the finding of terminally exhausted PD-1 + Tim-3 + T cells in a mouse model of B-cell tumors [7].

## TIM-3 blockade for AML and MDS immunotherapy

The first anti-TIM-3 antibody, sabatolimab (MBG453), demonstrated safety and efficacy in a phase I/Ib clinical trial (NCT02608268) in advanced solid tumors. In the STIMULUS clinical trial (NCT03066648), 53 patients with very high risk/high risk (vHR/HR)-MDS and 48 patients with newly diagnosed (ND) AML were treated with sabatolimab plus hypomethylating agent (HMA). A higher overall response rate (ORR) and 1-year progression-free survival (PFS) were shown for 51 vHR/HR-MDS and 40 ND-AML patients (Table 1). At present, 3 of 11 clinical studies involving TIM-3 inhibitors in AML/MDS have completed recruitment (NCT03066648, NCT03946670, and NCT04266301). In ASH2022, primary results from the ongoing STIMULUS-MDS1 (NCT03946670) were reported [8]. This trial is a randomized, double-blind, placebo-controlled, Ph II study of sabatolimab + HMA in patients with intermediate risk (IR), HR, or vHR-MDS who were ineligible for intensive chemotherapy or hematopoietic stem cell transplantation at screening. A total of 127 patients were randomized to either sabatolimab + HMA or placebo + HMA (Table 1). The primary complete remission (CR) rate was 21.5% vs. 17.7%, respectively, for these two groups. The CR + partial remission (PR) + hematologic improvement (HI) was 49.2% (sabatolimab + HMA) vs. 37.1% (placebo + HMA), respectively. In addition, the ongoing Ph III STIMULUS-MDS2 trial, which has a primary endpoint of overall survival (OS) (NCT04266301), has completed accrual. Another clinical trial of sabatolimab combined with an oral HMA drug (NCT04878432) is underway. Overall, sabatolimab + HMA was associated with a favorable safety profile in patients with HR-MDS [9].

**Table 1:** The efficacy of sabatolimab in the treatment of AML and MDS

Clinical trial identifier	NCT03066648				NCT03946670				
Phase	Ib				IIb				
Interventions	MBG453 + HMA				MBG453 + HMA		Placebo + HMA		
Cancer type (population, N)	vHR/HR-MDS(N = 51)	MDS with TP53	ND-AML(N = 40)	AML with TP53/RUNX1/ASXL1	IR/HR/vHR-MDS (N = 65)	MDS (BM blasts < 10%,N = 32)	IR/HR/vHR-MDS (N = 62)		MDS (BM blasts < 10%, N = 29)
mDOR of CR (month)	21.5		23		18.0		9.2		
ORR rate (%)	56.9	71.4	40	53.8	/				
mDOR (month)	16.1	21.5	12.6	12.6	/				
1 year PFS (%)	51.9		27.9		/				
mPFS (month)	/				11.1	11.3	8.5	8.3	
CR rate (%)	/				23.1	28.1	21.0	17.2	
CR + PR + HI rate (%)	/				49.2	53.1	37.1	34.5	
M (CR + PR + HI) (month)	/				13.4		9.2		
OS (month)	/				19.0		18.0		

**Notes**: AML, acute myeloid leukemia; BM, bone marrow; CR, complete remission; HMA, hypomethylating agent; IR, intermediate risk; MDS, myelodysplastic syndromes; mDOR, median duration of response; ND, new diagnosis; ORR, overall response rate; OS, overall survival; PFS, progression-free survival; PR, partial remission; vHR/HR, very high risk/high risk

In contrast, TIM-3-CD28 fusion proteins that turn inhibitory signals derived from TIM-3 engagement into activation by CD28 have been designed. These fusion proteins alone demonstrate the strongest response upon stimulation with anti-CD3 antibodies. Importantly, these proteins can increase the proliferation, activation, and cytotoxic capacity of conventional anti-CD19 CAR T cells [10]. Thus, combining IC fusion proteins with anti-CD19 CARs has the potential to increase the T cell proliferation capacity. In 2021, Lee et al. designed second-generation anti-TIM-3 CAR-T cells that exhibit potent anti-AML activity, including primary LSCs. Thus, anti-TIM-3 CAR-T cell therapy might be considered following first-line therapy to eradicate the LSCs present in minimal residual disease.

## Targeting the TIM-3 ligand Gal-9 for HM immunotherapy

Targeting the TIM-3 ligand galectin-9 (Gal-9) was recently considered as a novel immunotherapy for HMs. LYT-200, a fully humanized IgG4 αGAL-9 antibody, has been well tolerated in a phase I clinical trial for solid tumors. In ASH2022, a study reported the efficacy of LYT-200 in vitro and in vivo in multiple HMs. Further study demonstrated that LYT-200 also has in vivo protection and survival benefit in murine models of T cell acute lymphoblastic leukemia (T-ALL) and AML [11]. In ASH2022, another investigation with single-cell omics analyses demonstrated that Gal-9 expressed on chronic lymphocytic leukemia (CLL) cells led to T cell exhaustion, and Gal-9 antibody treatment of the CLL mouse model reduced disease development. These findings further supported that Gal-9 can be a novel immunotherapy target for CLL [12].

In summary, targeting TIM-3 has dual effects for directly inhibiting leukemia cells and restoring T cell activation. In addition, Gal-9 may serve as a novel immunotherapeutic target. Several targeted agents against TIM-3 are at different stages of development (Table 2).

**Table 2:** Targeted agents against TIM-3

type	products	mechanism	phase	disease or cell line	reference
Anti-TIM-3 antibody	sabatolimab (MBG453)	reverse T-cell exhaustion and anti-myeloid leukemia cell proliferation	Clinical Ib	vHR/HR-MDS, ND-AML	Brunner AM, et al. Blood. 2021
			Clinical II	IR/HR/vHR-MDS	Zeidan AM, et al. Blood. 2022
			Clinical III	IR/vHR/HR-MDS, CMML	Santini V, et al. Blood. 2022
TIM-3-CD28 fusion proteins	TIM-3/CD28-5 and TIM-3/CD28-6	increase proliferation of CAR-T cells, enhance cytokine secretion, proliferation of T cell	preclinical	K562 cell line	Blaeschke F, et al. Front Immunol. 2022
TIM-3-CAR-T cells	T3/28-CD19-CAR-T cells	enhance CAR-T cytotoxicity, cytokine secretion and persistence	preclinical	B-cell lymphoma	Zhao S, et al. J Immunother Cancer. 2021
	Anti-TIM-3 CAR-T cells	eradicate primary TIM-3 + CD34 + LSCs and sparing the TIM-3-CD34 + cells	preclinical	AML	Lee WS, et al. Mol Cancer Ther. 2021
Anti-GAL-9 antibody	aGal-9 antibody	reverse T-cell exhaustion	preclinical	CLL	Llaó Cid L, et al. Blood. 2022
	LYT-200	increase the ability to kill leukemia cells	preclinical	B/T-ALL, AML and DLBCL	Henry CJ, et al. Blood. 2022

**Notes**: AML, acute myeloid leukemia; CLL, chronic lymphocytic leukemia; CAR, Chimeric antigen receptor; DLBCL, diffuse large B cell lymphoma; GAL-9, galectin-9; IR, intermediate risk; LSCs, leukemia stem cells; MDS, myelodysplastic syndromes; ND, newly diagnosed; B/T-ALL, B/T cell-acute lymphoblastic leukemia; TIM-3, T cell immunoglobulin domain and mucin domain-3; vHR/HR, very high risk/high risk

## Data Availability

The material supporting the conclusion of this study has been included within the article.

## Abbreviations

AML: acute myeloid leukemia
AEs: adverse events
BiTE: Bispecific T-cell engagers
BCMA: B-cell maturation antigen
CLL: chronic lymphocytic leukemia
CAR: Chimeric antigen receptor
GAL-9: galectin-9
HMs: hematological malignancies
HI: hematologic improvement
IC: Immune checkpoint
IR: intermediate risk
LSCs: leukemia stem cells
MDS: myelodysplastic syndromes
ND: newly diagnosed
ORR: overall response rate
PFS: progression-free survival
PR: partial remission
RRMM: relapsed/refractory multiple myeloma
T-ALL: T cell-acute lymphoblastic leukemia
TIM-3: T cell immunoglobulin domain and mucin domain-3
vHR/HR: very high risk/high risk
